# The clinical and economic burden of obesity in low- and middle-income countries: a systematic review

**DOI:** 10.1038/s41366-025-01913-3

**Published:** 2025-09-29

**Authors:** Francis Fatoye, Chidozie Mbada, Faatihah Niyi-Odumosu, Clara Fatoye, Ushotanefe Useh, Zalmai Hakimi, Tadesse Gebrye

**Affiliations:** 1 Department of Health Professions, Manchester Metropolitan University, Birley Fields Campus, Manchester, UK; 2 Lifestyle Diseases, Faculty of Health Sciences, North‒West University, Potchefstroom, South Africa; 3 College of Health Science and Society, University of the West of England, Bristol, UK; 4 Sobi AB, Stockholm, Sweden

**Keywords:** Health care, Risk factors

## Abstract

Obesity has emerged as a critical public health challenge globally, with substantial health and economic repercussions. This study aimed to evaluate the literature on the clinical and economic burdens associated with obesity, specifically in low- and middle-income countries (LMICs). A systematic review following the Preferred Reporting Items for Systematic Reviews and Meta-Analyses (PRISMA) guidelines was performed. The CINAHL, MEDLINE, PubMed, Web of Science and Scopus databases were systematically searched for studies published from inception to March 28, 2025. The costs of illness for all included studies were converted to 2024 United States (US) dollars, using country-specific gross domestic product inflators. Conversion to US dollars was based on purchasing power parity (PPP). The quality of all included studies was assessed via the Newcastle‒Ottawa Scale (NOS). Of the total of 676 reports identified by the search strategy, six studies were prevalence-based, four studies were survey-based, and three model-based studies (n = 13) were eligible for inclusion on the basis of predefined inclusion criteria. These studies published data from Brazil, Ghana, China, Iran, South Africa, Mexico, and Thailand. Three of the 13 studies reported indirect costs. Two studies reported the clinical impact of obesity. Methodological quality was deemed moderate. The annual direct and indirect costs associated with obesity for a population in LMICs ranged from USD 0.2 billion to USD 12.56 billion and USD 223 million to USD 227.5 million, respectively. Hospitalisation was the main cost driver in five of the included studies. One study reported the total number of hospitalisations/number of person-years for men and women as 803/9207 and 2354/25,173, respectively. This is the first systematic review to summarise the clinical and economic burdens associated with obesity in LMICs. The clinical and economic burden of obesity on individuals and healthcare systems is significant, necessitating effective prevention and management strategies. To increase the accuracy and comparability of findings, future research should adopt a standardised cost-of-illness methodology. This approach will provide clearer insights into the economic impact of obesity and facilitate more effective public health interventions.

## Introduction

The World Health Organization (WHO) classifies obesity as a chronic, multifactorial disease characterized by excessive fat accumulation that presents a risk to health, operationalized as a body mass index (BMI) of 30 kg/m² or higher [1]. This metric is calculated by dividing an individual’s weight in kilograms by the square of their height in meters [2]. Obesity ranks as the fifth leading cause of mortality globally and is a major contributor to the rise of non-communicable diseases (NCDs) such as cardiovascular disease, type 2 diabetes, and certain forms of cancer [3]. Although obesity is often associated with high-income nations, it is increasingly prevalent across low- and middle-income countries (LMICs), where the shift from traditional to modern dietary patterns, combined with urbanization and sedentary lifestyles, is accelerating the public health crisis [4].

In 2014, the WHO estimated that over 600 million out of approximately 1.9 billion were people with obesity, with eight of the ten countries most affected being LMICs, including Brazil, China, Egypt, India, Indonesia, Mexico, Pakistan, and Russia [5, 6]. LMICs are nations classified by the World Bank based on gross national income (GNI) per capita, currently defined as those with a GNI per capita of $13,845 or less in 2024 (World Bank, 2024). Notably, 62% of the world’s population with obesity resides in LMICs [6]. Over the past three decades, age-standardised obesity rates have increased significantly, increasing from 3.2% to 10.8% in men and from 6.4% to 14.9% in women [7]. Specifically, in LMICs such as India, Bangladesh, and Nepal, the prevalence of obesity among women increased from 10.6% to 14.8%, from 2.7% to 8.9%, and from 1.6% to 10.1%, respectively, between 1996 and 2006 [8]. Interestingly, recent studies suggest that the prevalence of obesity in LMICs is lower than that in high-income countries [9].

Historically, undernutrition has dominated the public health discourse in LMICs. However, many of these countries are now experiencing a rapid epidemiological transition, where underweight and micronutrient deficiencies coexist with overnutrition and diet-related NCDs [10]. Urbanization, aggressive marketing of ultra-processed foods, declining physical activity, and increasing substance use such as tobacco and alcohol are driving this shift [11]. As a result, conditions like obesity, hypertension, and diabetes are emerging more rapidly in LMICs than in high-income settings. For example, cardiovascular diseases now account for a significant share of mortality in Ethiopia and Kenya, often linked to overweight and obesity [12].

Although obesity rates have started to stabilize in some high-income countries, this trend is not evident in the majority of LMICs, where rates continue to rise. Notably, urban areas in Rwanda, Zambia, and Brazil have shown renewed surges in obesity prevalence after initial periods of stabilization [13]. Among adolescents in LMICs including those in Egypt, Mexico, Vietnam, and Nigeria obesity rates are increasing at alarming rates [13]. A 2021 study spanning seven LMICs reported a consistent rise in overweight among adolescents, highlighting a troubling trajectory that portends a future wave of adult obesity and associated health complications [14]. Without comprehensive prevention programs, this trend is expected to continue, albeit at a potentially slower pace. However, even modest increases in obesity among youth populations can translate into significant public health costs in the long term, given the chronic nature of obesity and its complications [15].

Obesity is associated with several chronic lifestyle conditions, including hypertension, diabetes, cancer, and heart disease [16, 17]. These conditions contribute to disability, significant healthcare expenditures, and increased mortality rates. Obesity is reported to reduce life expectancy by an average of 5–8 years for women and 13–20 years for men [16]. Additionally, it is linked to various health issues, such as pain, fatigue, sleep disorders, and depressive moods [18]. Studies estimate that obesity can reduce life expectancy by 5–8 years in women and 13–20 years in men [19]. Moreover, obesity is associated with physical limitations such as chronic fatigue, sleep disturbances, joint pain, and depressive symptoms. For example, in Nigeria, patients with obesity undergoing cardiac surgery had significantly longer hospital stays compared to their non-obese counterparts, placing strain on already limited healthcare infrastructure [20]. Across LMICs, obesity-related disability-adjusted life years (DALYs) have been on a steady rise since 1990, and projections suggest they could double over the next four decades without effective intervention [21].

Despite the growing burden of obesity in LMICs, there is a lack of comprehensive synthesis evaluating its clinical and economic impacts in these settings. Although multiple studies have estimated the burden of obesity globally, significant variability in methods such as diagnostic criteria, cost components, population selection, and analytical approaches has led to inconsistent findings [22]. Most existing systematic reviews are either outdated, limited to high-income countries, or lack rigorous methodological assessments, such as quality appraisal, standardized cost conversions, or inclusion of both direct and indirect costs [22–25]. Furthermore, few reviews have focused specifically on LMICs, despite the rapidly increasing prevalence and unique contextual factors in these countries. Given the double burden of malnutrition and non-communicable diseases now faced by LMICs, a systematic review focused on these countries is urgently needed.

## Methods

A systematic review was conducted in accordance with the Preferred Reporting Items for Systematic Reviews and Meta-Analyses (PRISMA) guidelines [26]. The review has been registered with PROSPERO, the International Prospective Register of Systematic Reviews, under the registration number CRD42022331948. This registration ensures transparency and helps prevent duplication of research efforts.

### Search strategy

The literature search in this systematic review used the CINAHL, MEDLINE, PubMed, Web of Science, and Scopus databases and included articles published from inception to the 28^th^ of March 2025. The search terms used were obesity, overweight, obesity, unhealthy weight, high BMI, cost, expense, affordability, financial burden, health care costs, healthcare resource utilisation, emergency department visit, physician visits, hospitalisation, LMICs, low-income countries, middle-income countries, developing countries, Africa, Asia, Latin America, South America, and Central America. In addition to these databases, hand searches of the references of the included studies were also performed. The terms were matched with terms in the Medical Subject Heading (MeSH) database. All references were downloaded to EndNote, and duplicates were removed. The search was performed independently by two reviewers (TG & CM) to avoid the presence of bias in the selection and exclusion of studies. Disagreements were resolved by discussion with a third reviewer (FF). The details of the searching strategy with key words and initial hits are provided in Appendix A to ensure reproducibility and transparency of the work.

### Inclusion and exclusion criteria

On the basis of the predefined eligibility criteria, the reviewers (TG & CM) identified publications independently to be included in this review. Discrepancies were solved with the agreement of the reviewers (FF). The inclusion criteria for the search were studies on economic consequences or burdens of obesity (e.g., healthcare costs, societal costs, and treatment costs), the WHO’s recommendations for the definition of obesity (BMI ≥ 30 kg/m^2^), studies on humans related to obesity, studies published in the English language, and studies conducted in LMICs. The exclusion criteria included studies not related to the economic consequences of obesity; studies that did not calculate the burden of obesity; studies that characterised full economic evaluations, reviews, meta-analyses, cost-effectiveness studies, letters, notes, editorials and conferences; reports; letters to the editors; comments; opinions; protocols; studies with insufficient methodological details; and studies not published in the English language. These criteria ensured a focused selection of studies relevant to the clinical and economic impact of obesity in LMICs.

### Data extraction, quality assessment and reporting

**Fig. 1:**
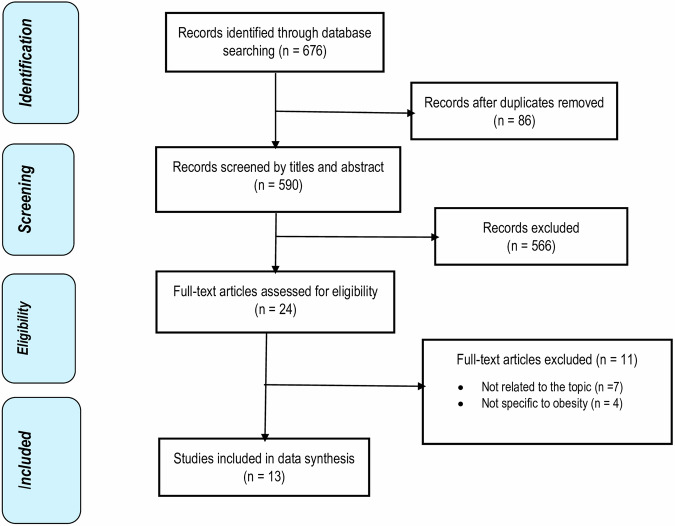
PRISMA flow diagram of publications included and excluded from the review.

The study selection process is illustrated in the PRISMA flow diagram (Fig. 1). Data extraction was performed by one reviewer (TG) and entered into a standardised data extraction table in Microsoft Excel. To ensure accuracy, the extracted data were independently verified by two additional reviewers (TG & CM). The following information was extracted from the selected studies: general details such as the first author and publication year, study country, type of cost analysis, sample size, socioeconomic status, BMI, study period, and study perspective. For cost-related data, we gathered information on the currency and cost year, along with mean or median total costs and costs attributable to obesity. The quality of the included studies was assessed via the Newcastle‒Ottawa Scale (NOS) by two reviewers (TG & CM). The NOS consists of nine items categorised into three dimensions: selection of the population, comparability of groups, and outcomes or exposures of interest [27]. Each study was scored on a scale with a maximum of nine points, where a score of ≥ 6 indicated high quality, 3 to 6 indicated moderate quality, and ≤ 3 indicated low quality. Any disagreements in scoring were resolved through consultation with a third reviewer (FF).

### Data analysis

In this study, summary descriptive statistics were utilised to outline the background and types of costs associated with obesity. Both healthcare and nonhealthcare costs were further analysed through quantitative methods. A comparison of costs across countries was conducted on the basis of the methodologies used in each included study. To facilitate these comparisons, costs were converted to US dollars (US$) via country-specific gross domestic product (GDP) deflators [28] and purchasing power parities (PPP) [29]. These conversions were performed as of August 2024. Estimated cost values were adjusted by multiplying them by the 2024 GDP coefficient, then dividing by the GDP of the reference year for each study, and finally adjusted by the PPP conversion factor for 2024. This approach ensured a standardised framework for comparing costs across different countries, taking into account economic variations.

## Results

The literature search retrieved 676 potentially relevant studies from PubMed (n = 167), Medline & CINHAL (n = 167), Scopus (n = 98) and Web of Science (n = 244) (see the PRISMA flowchart in Fig. 1). Of these, 182 were duplicates. After screening the titles and abstracts, 452 publications were excluded, leaving 42 articles for further full-text review. Thirteen studies met the inclusion criteria and were included in the review. The studies included in this systematic review were deemed to be of moderate quality (Appendix B).

### Characteristics of the included studies

The characteristics of each included study are detailed in Table 1. The studies reported data from various countries: Brazil (n = 6), Ghana (n = 1), China (n = 2), South Africa (n = 1), Mexico (n = 1), Iran (n = 1), and Thailand (n = 1). Among the 13 studies, three [30–32] were conducted from a societal perspective, whereas the remaining studies [33–40] focused on the health system perspective. Additionally, two studies [41, 42] specifically reported the clinical impact of obesity. Economic impact was assessed via different methodologies: prevalence-based [32, 33, 35, 37, 41], survey-based [30, 31, 36, 39], and modelling [34, 38, 40]. These studies were published between 2012 and 2022 and adopted varying time horizons, ranging from 6 months to 50 years.

**Table 1:** Characteristics of the studies included in the review.

Study	Country	Type of COI analysis	Patient population	Socio economic status	BMI, 30 kg/m^2^	Time horizon	Perspective	Outcomes measures
Kudel et al. [30]	Brazil	Survey	Obesity I (BMI 30 to <35) (n = 4423); Obesity II (BMI 35 to <40) (n = 1269); Obesity III (BMI 40 + ) (n = 707) Brazilian adults, aged 18 years and older.	Obesity I (high school or less (n = 35.4%), at least some college (64.6%) Obesity II (high school or less (n = 36.3%), at least some college (n = 63.7%) Obesity III (high school or less n = 40.6%, at least some college n = 59.4%)	Obesity class I (BMI, 30–34.99), Obesity class II (BMI, 35–39.99) and Obesity class III (BMI, 40 + )	N/A	Societal perspective	Direct costs and indirect costs
Bahia et al. [33]	Brazil	Prevalence-based	54,339, (M = 20,764, W = 33,575) Aged ≥18 years	N/A	≥30	2008 to 2010 (3 years)	Health System	Direct costs
Sichieri et al. [41]	Brazil	Prevalence-based burden-of-obesity approach	Total = 652 (W = 516 M = 136) ‘people with obesity’ between 20 to 60 years of age	N/A	≥30	2001	N/A	Average length of stay
Lartey et al. [34]	Ghana	Model-based	Older adults who were 50 years and above	N/A	≥ 30.0	50-year time horizon	Health system and patient perspectives	Direct healthcare costs

Li et al. [31]	China	Cross-sectional (community-based)	337 participants (M = 174, W = 163)	Illiterate (14.5%) Primary (grades 1 to 6) (23.6%) Middle (grades 7 to 9) or higher (62%)	≥30	1 year	Societal perspective	Direct costs and indirect costs
Boachie et al. [35]	South Africa	Prevalence-based approach	28,000 The age group 15–24 years The sample size for people with obesity	N/A	≥30	1 year	Health system	Direct health care cost
Ramezankhani et al. [42]	Iran	Questionnaire interviews	2210 people with obesity (73% of Women)	Marital status (n) Single (M = 16, W = 31) Married (M = 580, W = 1348); Widowed (M = 5, W = 230) Educational level (%) <6 years (M = 34.1, W = 57.1); 6–12 (M = 52.2 W = 38.7) ≥ 12 (M = 13.6, W = 4.2)	M = 32.4; W = 33.6	1999–2018	N/A	Hospitalisations per follow-up time
Shi et al. [36]	China	The China Health and Retirement Longitudinal Study surveys	13,323 adult individuals (obesity = 1500) Male 34.81%; Female 65.02% Mean (SD) age = 58.15 (9.01) years Response rate of 80.5%.	No university degree= 97.21% University degree 2.79% Smoking status (Never = 71.66%; Quit = 2.02%; Still have = 16.27%; Missing 0.05%) Standard of living (Relatively poor or poor = 40.37%; Average 52.07%; High or relatively high = 5.62%)	≥30	Between June 2011 and March 2012	Health system	Direct health care costs

de Oliveira et al. [37]	Brazil	Top-down approach based on prevalence	Data from 55,970 households and 188,461 respondents	N/A	≥30	Between 2008 and 2009	Health system	Direct healthcare cost
Rtveladze et al. [38]	Brazil	Model based	In 2010, the model estimates that 16% male and 14% female population were people with obesity and obesity related. This is predicted to increase in 2050 to 46% and 20%, respectively.	N/A	≥30	Between 2010 and 2050	Health system	Direct healthcare cost
Canella et al. [39]	Brazil	Household Budget Survey	Population-based study involving 55,970 Brazilian households Proportion of women in family unit (%) = 51.8	Monthly household income per capita (US$) = 644.70	≥30	May 2008 and May 2009	Health system	Direct healthcare cost
Rtveladze et al. [40]	Mexico	Model based (Microsimulation)	Mexican Health and Nutrition Surveys 1999 and 2000	N/A	≥30	Between 2010 and 2050	Mexican National Health and Nutrition Survey 2006	Direct healthcare cost
Pitayatienanan et al. [32]	Thailand	Prevalence-based	The database of the Centre for Health Equity Monitoring (CHEM), Faculty of Medicine, Naresuan University. outpatient The Central Office for Health care Information (COHI) database, 2009…inpatient	N/A	≥30	2009	Societal perspective	Direct costs and indirect costs

*BMI* body mass index, *W* women, *M* men, *N/A* not available, *COI* cost of illness, *SD* standard deviation.

### Summary of findings

The key findings from the included studies are summarised in Table 2. The annual costs related to obesity in low- and middle-income countries (LMICs) are estimated to be between US$ 544 million and USD 12.56 billion for direct expenses and between USD 203 million and USD 227.5 million for indirect expenses. In Brazil, hospitalisation costs for individuals with class III obesity were found to be twice as high, with indirect costs nearly double those of individuals with a normal BMI [18]. Specifically, the annual physician and hospitalisation costs for class III obesity patients were reported at USD 188.14 and USD 1,839.70, respectively. In Ghana, a cost of illness study estimated that the annual direct healthcare costs for female and male patients were USD5,530 and USD4923, respectively [22]. Moreover, in rural Yunnan Province, China, the direct cost for individuals aged 35 years and older was estimated at USD 3.774 billion, whereas the indirect cost was estimated at USD 203 million [19]. These findings highlight the substantial economic burden of obesity across different LMICs. Three studies from Brazil, China, and Thailand estimated the indirect costs associated with obesity [18–20]. In Brazil, the average annual indirect cost estimate for individuals with class III obesity is USD 970.00 [18]. This study utilised the Work Productivity and Activity Impairment-General Health Questionnaire, a validated instrument that measures absenteeism, presenteeism, overall work productivity loss, and activity impairment. In China, the indirect cost related to obesity is estimated at USD 203 million [19], whereas in Thailand, it is estimated at USD 227 million [20]. Table 3 summarizes the cost components included across the studies. Hospitalisation emerged as the primary cost driver in six of the included studies [18–21, 23, 24]. In Brazil, the average length of hospital stay for obesity-related diseases was reported to be 7.9 days for men and 6.8 days for women [29]. The burden of hospitalisation associated with obesity was notably substantial in both Brazil [29] and Iran [30], underscoring the need for effective management strategies to address this issue.

**Table 2:** Summary of findings—direct costs, hospitalisation rates and indirect costs.

Study	Year of costing	Main findings (costs inflated 2024 US$)	Comments
Kudel et al. [30]	2015	**Physician costs** (obesity class I, US$159.8) and obesity class II (US$180.34), obesity class III (US$188.14)) **Hospitalisation costs** (obesity class I (US$840.2), obesity class II (US$933.9), and obesity class III (US$1839.7)) **Indirect cost** (obesity class I (US$582), obesity class II (US$867.8) and obesity class III (US$970.2)	Hospitalisation costs were over twice as high and indirect costs were nearly double for obesity class III than for normal BMI respondents.
Bahia et al. [33]	2010	Hospitalisation costs = US$ 1566.9 million); Ambulatory costs (medical visits, exams, procedures) = US$ 701.1 million; **Total costs** = US$ 2.2 billion	Obesity attributable hospitalisation costs were higher among men, although population-attributable risk (PAR) was lower than in women. The inverse situation was seen regarding outpatient costs, with much higher PAR but lower costs among men.
de Oliveira et al. [37]	2011	Direct costs attributable to obesity, US$ 544 million The cost of morbid obesity, US$ 129.5 million; Bariatric surgery costs in Brazil total US$ 35.11 million	The cost of morbid obesity in women was five times higher than it was in men. The highest costs attributable to morbid obesity were for ischemic heart disease and diabetes.
Pitayatienanan et al. [32]	2009	The direct health care cost attributable to obesity was estimated at US$193.3 million or 1.5% of national health expenditure. The cost of productivity loss attributable to obesity was estimated at US$227.5 million- accounting for 54% of the total cost of obesity.	Costs associated with health care provision and costs associated with productivity loss were broadly similar.
Canella et al. [39]	2009	Monthly household expenses on medicines per capita in households with obesity was US$ 46.8 Public Sector expenses per capita (obtained in the SUS) = US$9.2 Private Sector (paid for out-of-pocket) per capita = US$37.1	Out-of-pocket expenses on medicines were always higher than the cost of medicines obtained through the public sector.
Sichieri et al. [41]	N/A	Average length of stay (days), M = 7.9; Women = 6.8 Hospitalisation rate attributable to obesity for men (37.5) and for women (130.8).	Diseases associated with obesity had a significant impact on hospitalisations and economic costs in Brazil.
Ramezankhani et al. [42]	N/A	Total number of hospitalisation/number of person-years (M = 803/9207, W = 2354/25,173	Obesity was associated with increased hospitalisation rates during long-term follow-up.
Lartey et al. [34]	2017	**Female:** Out of Pocket Costs = US$ 2,021.2 National Health Insurance = US$3,508 Total costs = US$5530 (95% CI: 5,493.6 to 5568) **Male**, Out of Pocket costs = US$1,801 (1785-1814.5) National Health Insurance = US$3,124 (3097.6–3148) Total costs = US$4,923 (4885 – 4963)	Obesity is associated with significantly higher healthcare costs, with these effects being higher in females.
Li et al. [31]	2015	Direct cost = US$3,774million Indirect costs = US$203 million	Direct expense comprised the biggest constituent of total costs, accounting for more than 90% of costs, and hospitalisation was the largest driver of direct medical costs. Males had higher direct costs and higher overall disease expenses than females.
Rtveladze et al. [38]	2010 & 2050	The health care costs will double from US$12.56 billion (2010) to US$23.8 billion (2050).	Relatively small reductions in the level of BMI can lead to substantial disease reduction and cost savings for the health care system.
Rtveladze et al. [40]	2000	Costs of US$1,445.3 million are estimated for 2010, projected to increase to US$ 2.15 billion and US$ 3.04 billion in 2030 and 2,050, respectively	A 1% reduction in BMI prevalence could save US$ 43 million in healthcare costs in 2030 and US$ 85 million in 2050.
Boachie et al. [35]	2020	Total costs per patients ranges US$1,763 million to US$2,085 million	The main driver of obesity cost (approximately 91%) were coming from hypertension and diabetes.
Shi et al. [36]	2011	Annual total direct health care costs = RMB 2,050.68) Annual outpatient costs (RMB 587.68) Annual inpatient costs (RMB 302.70) Annual self-health care costs (RMB 1,160.31)	Patients with obesity had significantly higher total direct health care costs compared with the normal-weight group.

*N/A* not applicable, *BMI* body mass index, *W* women, *M* men, *US$* United States dollar, *Bht* the official currency of Thailand, *RMB* official currency of the People’s Republic of China, *ZAR* currency of South Africa.

**Table 3:** Cost components included.

	Kudel et al. [18]	Bahia et al. [21]	Lartey et al. [22]	Li et al. [19]	Boachie et al. [23]	Shi et al. [24]	Rtveladze et al. [26]	de Oliveira et al. [25]	Canella et al. [27]	Rtveladze et al. [28]	Pitayatienanan et al. [20]
Direct cost								Y		Y	
Medicine					Y	Y			Y		
Chemotherapy administration, radiation; laparoscopic cholecystectomy; physiotherapy, speech-therapy					Y						
Laboratory					Y						
Scan and Imaging					Y						
Physician	Y	Y									
Hospitalisation	Y	Y		Y	Y	Y					Y
Ambulatory cost		Y						Y			
National Health Insurance			Y								
Out of pocket			Y	Y							
Hospital admissions (inpatient care)								Y			
Outpatient visits					Y	Y		Y			Y
Diagnostic tests				Y							
Transportation				Y							
Accommodation costs of the patient and family members during these visits and the expenditures of caregivers				Y							
Indirect costs											
Productivity loss related to morbidity, including expense resulting from absence from work and/or difficulty with paid employment due to morbidity.	Y			Y							Y

*Y* yes.

## Discussion

This systematic review provides valuable insights into the clinical and economic impact of obesity in LMICs, highlighting significant variations in costs across different nations. The review identified only 13 studies that met the inclusion criteria, indicating a limited but crucial body of literature on this topic. The estimated annual costs associated with obesity, including both direct and indirect expenses, varied widely from US$544 million to US$12.56 billion for direct costs and US$ 223 million to US$227.5 million for indirect costs. These figures emphasise the substantial economic burden of obesity, with hospitalisation identified as the primary cost driver. One critical point is the heterogeneity among the studies, stemming from differences in methodological approaches, perspectives, and target populations. Beyond these high-level factors, heterogeneity also appeared to arise from variations in how obesity was defined and measured across studies, the types of cost components included, and the timeframes over which costs were assessed. Additionally, some studies employed national datasets while others relied on sub-national or hospital-level data, leading to differences in representativeness and scale. Economic modelling techniques and assumptions such as discount rates, cost inflation adjustments, and currency conversions also varied, further complicating cross-study comparisons [43]. These methodological disparities underscore the challenge of synthesising findings across settings and reinforce the need for unified costing frameworks to better inform policy responses in LMICs.

This systematic review aligns with the literature indicating that individuals with obesity experience significantly greater healthcare resource utilisation than those with a normal BMI [44, 45]. The current review highlights the multifaceted costs of obesity, including humanistic impacts such as binge eating, anxiety, and depression, as well as societal costs linked to lost productivity, which encompasses both directly missed workdays and the reduction of future earnings due to morbidity and mortality [30–32]. Despite limited discussion across studies, productivity costs particularly in LMICs represent a substantial but often underreported component of the economic burden. The studies included in this review employed the human capital approach, which estimates productivity loss by assigning a monetary value to time lost from work due to illness or premature death. This method typically involved calculating missed workdays multiplied by the average daily wage for morbidity-related losses and projecting lost future income for mortality-related losses, adjusted with discount rates. However, assumptions varied across studies regarding the inclusion of caregiver absenteeism, time per outpatient visit, and wage metrics, contributing to variability in estimates. The underrepresentation of broader productivity impacts, such as reduced job performance and long-term employment limitations, suggests a need for more comprehensive methodologies. Given these findings, it is crucial for public health authorities to prioritise preventive interventions aimed at reducing obesity. Strategies should focus on promoting physical activity and encouraging healthy lifestyle choices [46]. These interventions could help reduce direct costs related to physician services, hospitalisation, and outpatient care, ultimately benefiting both individuals and the wider economy [22]. This comprehensive approach can help address the growing obesity epidemic and its associated burdens in LMICs.

Dee et al. [47] found that, in high-income countries, the indirect costs of overweight and obesity such as lost productivity tended to surpass direct medical expenses. However, in contrast, two studies included in the current review [30, 31] found that direct healthcare costs related to obesity were greater than the indirect costs. Notably, only a few studies in this review [30–32] considered indirect costs, suggesting that the full economic burden from productivity losses may be underestimated. Additionally, while Dee et al.’s findings were based on high-income settings, the present review focuses on LMICs, where estimating indirect costs is particularly challenging. Many individuals in LMICs are employed in the informal sector, where income is often unstable and difficult to measure [48, 49]. Furthermore, cultural perceptions of obesity may influence healthcare-seeking behaviors and the distribution of health resources, potentially leading to higher direct spending [50]. These contextual factors may help explain why, in LMICs, direct costs appear to outweigh indirect ones.

In this systematic review, five studies employed a prevalence-based approach to estimate the costs associated with obesity [32, 33, 35, 37, 41]. This method allows for the estimation of costs incurred over a specified period, measuring the economic burden of obesity without considering when the condition first developed [51]. While prevalence-based cost-of-illness analyses provide valuable insights, it is essential to acknowledge potential limitations. The reported cost estimates may not fully reflect the current economic impact or be entirely applicable to the specific population under study. This variability highlights the need for caution when interpreting the results, as the estimates may lack accuracy and timeliness. Future research should consider incorporating more dynamic modelling approaches that account for the temporal aspects of obesity-related costs to improve the precision and relevance of findings.

The variation in healthcare systems across countries poses a critical challenge to generalising obesity cost estimates, as most studies in this review offer country-specific data that reflect distinct healthcare infrastructures and economic conditions within LMICs. This limits the transferability of findings across settings and underscores the need for robust multinational studies. The World Obesity Atlas exemplifies such efforts, providing a globally harmonised model that estimated the economic impact of overweight and obesity at US$1.96 trillion in 2020, or 2.4% of global GDP, with projections rising to nearly 3% by 2035 [52]. These figures capture both direct and indirect costs, including productivity losses and premature mortality, and highlight the disproportionate burden on LMICs, which are expected to house two-thirds of adults with severe obesity by 2030 [43]. This disparity reveals the limitations of isolated national studies and demonstrates the value of cross-country analyses like those used in the World Obesity Atlas and Global Burden of Disease project. Integrating such global models with local data enables more accurate forecasting, promotes international benchmarking, and supports the development of context-specific, scalable interventions ultimately enhancing the global response to the growing obesity epidemic.

This systematic review is the first to comprehensively assess both the clinical and economic burden of obesity in LMICs, offering valuable and policy-relevant insights into an underexplored area of global health. The study followed a structured and transparent methodology, drawing from five major databases; however, this may have limited the inclusion of relevant studies indexed elsewhere [53]. The exclusion of non-English publications could also have led to the omission of important research from non-English-speaking LMICs. Significant heterogeneity in study designs, outcome measures, and costing approaches prevented meta-analysis and highlighted the urgent need for standardized reporting guidelines in obesity research. Only two [41, 42] of the thirteen included studies focused on clinical outcomes, restricting insights into the broader health impacts of obesity. Moreover, the reliability of cost estimates is constrained by the generally moderate to low quality of included studies, as assessed using the Newcastle-Ottawa Scale. In spite of these limitations, we believe that this review was systematic in nature and summarizes all available and relevant clinical and economic burden results from the literature.

## Conclusion

This is the first systematic review to summarise the clinical and economic burdens associated with obesity in LMICs. The clinical and economic burden of obesity on individuals, families, healthcare systems and society is significant. In addition, obesity in LMICs is associated with significant direct healthcare costs. These findings underscore the need for effective prevention and management strategies to reduce the associated burden. However, the studies included in this review utilised diverse approaches, and many presented methodological shortcomings related to resource use measurement and cost allocation. To increase the validity and comparability of findings, future research should adopt a standardised cost-of-illness methodology. This would help ensure more accurate assessments of the economic impact of obesity, facilitating evidence-informed decision-making for public health interventions. By addressing these methodological challenges, we can better understand the true burden of obesity and develop more effective strategies to mitigate its impact.

## Supplementary information

Search strategy and study quality assessment
