# Supplementary material for: The clinical and economic burden of obesity in low- and middle-income countries: a systematic review
Source: Int J Obes (Lond). 2025 Sep 29;49(12):2453–61. doi: 10.1038/s41366-025-01913-3 (PMC12634439; doi:10.1038/s41366-025-01913-3)
Supplement: Supplementary file 1 — Search strategy and study quality assessment [file 41366_2025_1913_MOESM1_ESM.docx]

**Appendix A**. Details of the Search Strategy with Keywords and Initial Hits:

**CINHAL & Medline**

S4 S1 AND S2 AND S3 …………… (167)

S3 AB (LMICs or low income countries or middle income countries or developing countries or Africa or Asia or Latin america or south america or central america) ………… (73,995)

S2 AB costs or cost or expense or affordability or financial burden or health care costs or healthcare resource utilisation or emergency department visit or Physician visits or hospitalization …………… (391,608)

S1 AB obesity or overweight or obese or unhealthy weight or high bmi ………. (122,432)

**PubMed**

#5 ((costs[Title/Abstract] OR cost[Title/Abstract] OR expense[Title/Abstract] OR affordability[Title/Abstract] OR financial burden[Title/Abstract] OR health care costs[Title/Abstract] OR healthcare resource utilisation[Title/Abstract] OR emergency department visit[Title/Abstract] OR Physician visits[Title/Abstract] OR hospitalization[Title/Abstract]) AND (AB obesity[Title/Abstract] OR overweight[Title/Abstract] OR obese[Title/Abstract] OR unhealthy weight[Title/Abstract] OR high bmi[Title/Abstract])) AND (LMICs[Title/Abstract] OR low income countries[Title/Abstract] OR middle income countries[Title/Abstract] OR developing countries[Title/Abstract] OR Africa[Title/Abstract] OR Asia[Title/Abstract] OR Latin america[Title/Abstract] OR south america[Title/Abstract] OR central america[Title/Abstract]) ……167

#4 LMICs[Title/Abstract] OR low income countries[Title/Abstract] OR middle income countries[Title/Abstract] OR developing countries[Title/Abstract] OR Africa[Title/Abstract] OR Asia[Title/Abstract] OR Latin america[Title/Abstract] OR south america[Title/Abstract] OR central america[Title/Abstract]….422,140

#3 obesity[Title/Abstract] OR overweight[Title/Abstract] OR obese[Title/Abstract] OR unhealthy weight[Title/Abstract] OR high bmi[Title/Abstract]….232,090

#1 costs[Title/Abstract] OR cost[Title/Abstract] OR expense[Title/Abstract] OR affordability[Title/Abstract] OR financial burden[Title/Abstract] OR health care costs[Title/Abstract] OR healthcare resource utilisation[Title/Abstract] OR emergency department visit[Title/Abstract] OR Physician visits[Title/Abstract] OR hospitalization[Title/Abstract]…..1,050,463

**Web of Science**

#4 #1 AND #2 AND #3 ………….244

#3 LMICs or low income countries or middle income countries or developing countries or Africa or Asia or Latin america or south america or central america (Abstract)…….927,529

#2 costs or cost or expense or affordability or financial burden or health care costs or healthcare resource utilisation or emergency department visit or Physician visits or hospitalization (Abstract)….2,640,508

#1 obesity or overweight or obese or unhealthy weight or high bmi (Abstract)……..472,919

**Scopus**

#4 ( ABS ( lmics or low income countries or middle income countries or developing countries or africa or asia or latin america or south america or central america ) ) and ( ABS ( costs or cost or expense or affordability or financial burden or health care costs or healthcare resource utilisation or emergency department visit or physician visits or hospitalization ) ) and ( ABS ( obesity or overweight or obese or unhealthy weight or high bmi ) ) Show less …..98

#3 ABS ( lmics or low income countries or middle income countries or developing countries or africa or asia or latin america or south america or central america )………2,856

#2 ABS ( costs or cost or expense or affordability or financial burden or health care costs or healthcare resource utilisation or emergency department visit or physician visits or hospitalization )….716

#1 ABS ( obesity or overweight or obese or unhealthy weight or high bmi )………74,301

**Appendix B: Study quality assessment**

**Appendix B1:** Quality assessment checklist for nonrandomized studies.

| **Domain** | **Checklist Criteria** | **Additional Information** |
| --- | --- | --- |
| **Selection** | 1. Is the case definition adequate?    1. yes, with independent validation *    2. yes, eg record linkage or based on self-reports    3. no description |  |
|  | 1. Representativeness of the cases    1. consecutive or obviously representative series of cases *    2. potential for selection biases or not stated | **Assumption: Given the nature of the review, studies received a star if they discuss representation (and reasons for their study being representative) or if they are multicentre/regional/national.** |
|  | 1. Selection of Controls    1. community controls *    2. hospital controls    3. no description | **Assumption: It was assumed that if the study was in a hospital setting in which cases were hospital patients, hospital controls were accepted.** |
|  | 1. Definition of Controls    1. no history of disease (endpoint) *    2. no description of source | **Assumption: History of disease/infection was used in this criteria even in studies looking at mortality or other burden outcomes** |
| **Comparability** | 1) Comparability of cases and controls on the basis of the design or analysis   1. study controls for age/sex/comorbidities * 2. study controls for any additional factor * | **2 * maximum allotted for this criteria.**  **Assumption: For studies in which hospital associated cases and LoS were being analysed, two stars were only given if time dependency was controlled for** |
| **Exposure** | 1. Ascertainment of exposure    1. secure record (eg surgical records) *    2. structured interview where blind to case/control status *    3. interview not blinded to case/control status    4. written self-report or medical record only    5. no description | **Assumption: Studies which utilised lab techniques were used to ascertain exposure received one star.** |
|  | 1. Same method of ascertainment for cases and controls    1. yes *    2. no |  |
|  | 1. **Non-Response rate**    1. **same rate for both groups ***    2. **non respondents described**    3. **rate different and no designation** | **Assumption: No description of data cleaning or linkage and loss to missing data for retrospective studies was panelised by not awarding a star** |

**Appendix B2:** Quality of the included studies.

| Study | Selection 1 * | Selection 2 * | Selection 3* | Selection 4 * | Comparability 1** | Exposure 1 * | Exposure 2 * | Exposure 3* | Total  (MAX. 9) |
| --- | --- | --- | --- | --- | --- | --- | --- | --- | --- |
| Kudel et al. [30] | 1 | 1 | 0 | 0 | 2 | 1 | 0 | 1 | 6 |
| Bahia et al. [33] | 1 | 1 | 0 | 0 | 2 | 1 | 0 | 0 | 5 |
| Sichieri et al. [41] | 1 | 1 | 0 | 0 | 0 | 1 | 0 | 0 | 3 |
| Lartey et al. [34] | 1 | 1 | 0 | 0 | 0 | 1 | 0 | 0 | 3 |
| Li et al. [31] | 1 | 0 | 0 | 0 | 2 | 1 | 0 | 1 | 5 |
| Boachie et al. [35] | 1 | 1 | 0 | 0 | 2 | 1 | 0 | 0 | 5 |
| Shi et al. [36] | 1 | 1 | 0 | 0 | 2 | 1 | 0 | 0 | 5 |
| de Oliveira et al. [37] | 1 | 1 | 0 | 0 | 2 | 1 | 0 | 0 | 5 |
| Rtveladze et al. [38] | 1 | 1 | 0 | 0 | 0 | 1 | 0 | 0 | 3 |
| Ramezankhani et al. [42] | 1 | 1 | 0 | 0 | 2 | 1 | 0 | 1 | 5 |
| Canella eta al. [39] | 1 | 1 | 0 | 0 | 0 | 1 | 0 | 1 | 4 |
| Rtveladze et al. [40] | 1 | 1 | 0 | 0 | 0 | 1 | 0 | 0 | 3 |
| Pitayatienanan et al. [32] | 1 | 1 | 0 | 0 | 2 | 1 | 0 | 0 | 5 |
